# Exosomes and Their Bioengineering Strategies in the Cutaneous Wound Healing and Related Complications: Current Knowledge and Future Perspectives

**DOI:** 10.7150/ijbs.80430

**Published:** 2023-02-27

**Authors:** Guang Yang, Saquib Waheed, Cong Wang, Mehdihasan Shekh, Zhibin Li, Jun Wu

**Affiliations:** 1 Department of Burn and Plastic Surgery, Shenzhen Institute of Translational Medicine, Shenzhen Second People's Hospital, The First Affiliated Hospital of Shenzhen University, Shenzhen 518035, China.; 2 Division of Nephrology, Peking University Shenzhen Hospital, Peking University, Shenzhen 518036, China.; 3 Department of Biomedical Engineering, School of Medicine, Shenzhen University, Shenzhen 518060, China.; 4 College of Materials Science and Engineering, Shenzhen University, Shenzhen 518060, China.; 5 Human Histology & Embryology Section, Department of Surgery, Dentistry, Pediatrics & Gynecology, University of Verona Medical School, Verona 37134, Italy.

**Keywords:** exosomes, stem cells, engineered exosomes, wound healing, biomaterials, complex wounds, complications, biomarkers.

## Abstract

Exosomes, as therapeutically relevant cell-secreted extracellular vesicles, have attracted enormous interest because they participate in intercellular communication and facilitate wound healing. Stem cell-derived exosomes exhibit similar biological effects to source cells with the exception of low immunogenicity and no tumorigenicity, as well as superior efficacy in promoting wound healing. Exosomes accelerate wound healing by promoting angiogenesis and cell proliferation, as well as balancing inflammatory responses. Particularly, when exosomes are genetically modified or used in combination with materials, they can exhibit better comprehensive therapeutic properties, such as enriching active ingredients, targeted delivery, and physiological barrier to penetration, which are not available in traditional single products. Besides, exosomes have also been considered for diagnostic and therapeutic uses related to wounds, such as repairing complex wounds, enhancing graft success, treating related complications, and serving as diagnostic biomarkers. However, their clinical applications still face challenges, as reliable commercial products are not yet available. This review will focus on recent research advances that describe the characteristics and isolation of exosomes, introduce the sources of exosomes suitable for wound repair and related complications, illustrate the value of engineered exosomes and their development directions in the future, and provide evidence for the potential therapeutic application of exosomes in wound healing, as well as discuss potential risks, challenges, and solutions for future applications.

## Introduction

Exosomes are extracellular vesicles (EVs) generally with about 30-150 nm diameter 1. Intraluminal vesicles are formed by the invagination of multivesicular bodies in the endosome-lysosomal pathway 2, 3. Under the action of vesicle transport-related proteins, multivesicular bodies containing intraluminal vesicles will fuse with the cell membrane, and the released intraluminal vesicles are called exosomes. Exosomes contain various proteins, nucleic acids, and essential cell communication mediators. Among them, stem cell-derived exosomes have similar biological functions to their sources while at the same time possessing the advantages of small size, easy penetration of biological membranes, low immunogenicity, easy storage, and no tumorization 2, 4. Their unique lipid bilayer membrane structure can protect its contents and resist environmental damage. The advent of stem cell preparation technology has rendered stem cells towards clinical translation. The industrialization of stem cell drugs is being promoted all over the world. However, the safety of stem cells is still the focus of the clinical application. Compared with stem cells, exosomes are considered safer biopharmaceuticals 5. Consequently, exosomes are regarded as a kind of biological medicine with the potential for development.

Exosomes have been viewed as "naturally domesticated" endogenous nanocarriers in the past few years. Recently, the rise of the concept of engineered exosomes has broken this notion, allowing exosomes to become “artificially domesticated” nanocarriers 6, 7. Nowadays, genetic or chemical engineering can modify exosomes to have specific biological functions. It improves the ability of exosomes to load drugs and presents excellent drug delivery properties, such as non-liver-targeted nucleic acid drug delivery and blood-brain barrier penetration. Additionally, exosomes can also be combined with a variety of materials. In addition to increasing the performance of the raw materials, some disadvantages can also be decreased. These characteristics make exosomes promising for wound healing.

Wound healing is a complex biological process involving many different stages. The wound treatment will be more complicated when the patient has other diseases. For example, burns cause skin injury and systemic complications, so medication should be used carefully. In wound healing, tissue regeneration mainly depends on the growth and proliferation of cells, and the existing drugs or growth factors provide an auxiliary effect. Several studies have shown that stem cells and their exosomes can benefit wound healing 8-11. In addition to promoting cell proliferation and tissue regeneration, exosomes also reduce scarring, complications, and immune responses to graft materials 12, 13. These multiple therapeutic effects are more attractive than single protein drugs. Moreover, the continuous modification of exosomes, such as genetic modification, chemical treatment, and binding materials, improves the therapeutic effect. Therefore, this review will describe the characterization, preparation, and origin of exosomes, focusing on the application of exosomes in conventional and complex wound healing, discuss the research progress of engineered exosomes in wound repair, and present directions for future research.

### Biological characteristics of exosomes

#### Exosomes and EVs

The release of EVs during the development of reticulocytes was first discovered in 1983 14. All secreted membrane vesicles are now referred to as EVs. Endocytic-derived vesicles with a diameter of 30 to 1000 nm are released into the extracellular environment by various cell types, including platelets, B cells, T cells, mast cells, cancer cells, and others 15-17. They can only be detected using fluorescently tagged fluorescence microscopy rather than standard light microscopy because they are typically 5- to 10-fold smaller than wavelengths of light visible. EVs can be found in various cell types and body fluids, including saliva, urine, blood and breast milk 18.

**Figure 1:**
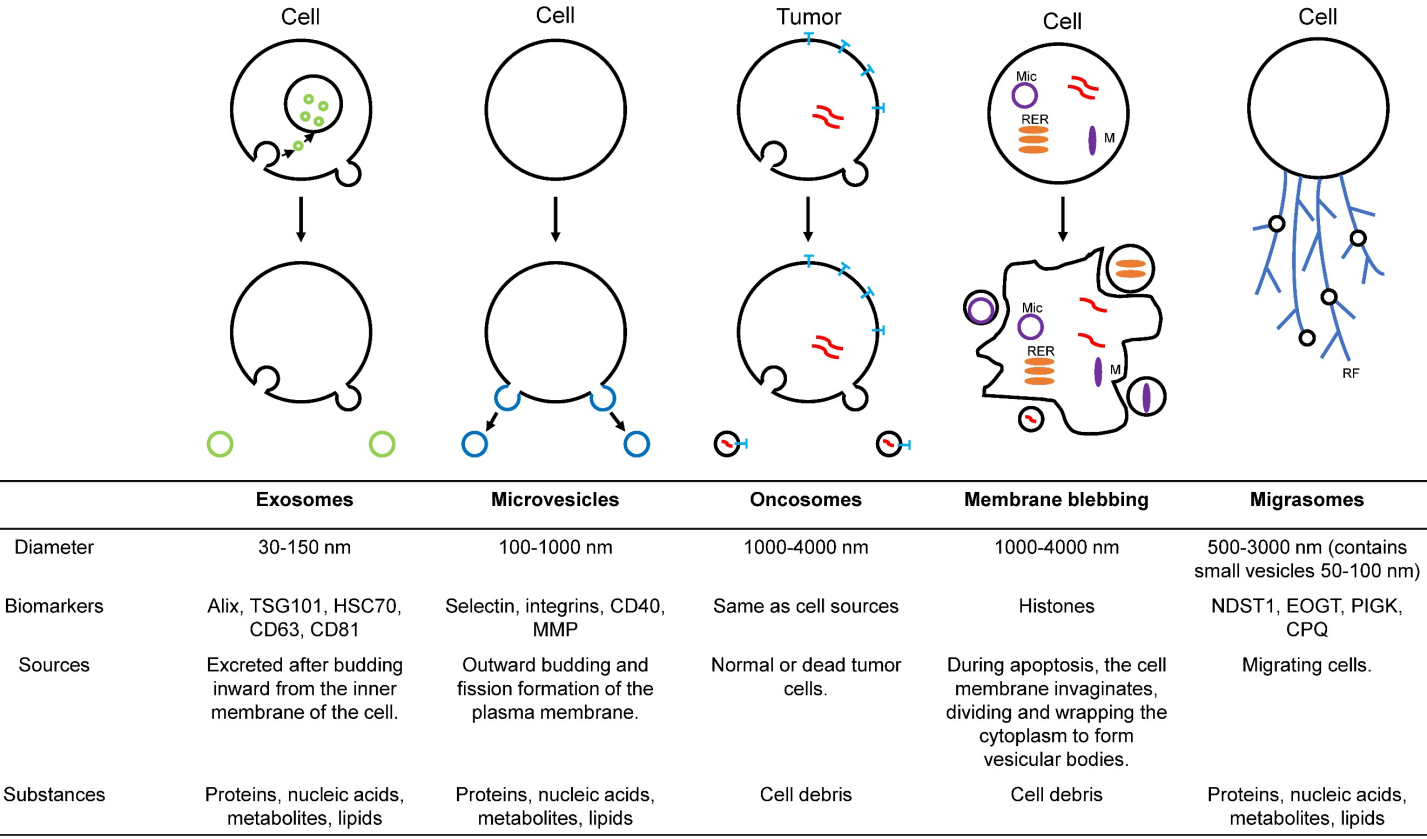
** Illustration of the major populations of extracellular vesicles. Exosomes are endosome-derived EVs formed by inward budding from the cell membrane.** Microvesicles arise from fission and outward budding of the plasma membrane. Oncosomes are membrane-derived extracellular vesicles secreted by cancer cells. Plasma membrane blebbing is the apoptotic or pyroptotic body produced by programmed cell death. Migrasomes originate from migrating cells. M = mitochondria, RER = rough endoplasmic reticulum, Mic = micronuclei, RF = retraction fibrils.

With an in-depth understanding of EVs, it has been found that EVs can also be divided into various subtypes according to diameter, function, and source (figure 1). Exosomes are a crucial subtype of EVs 19. These vesicles have been shown to have intrinsic biological properties with essential roles in cell-to-cell communication and regulation of cell function, with the potential to be novel therapeutic agents. Microparticles/microvesicles are released directly from the plasma membrane and are approximately 100-1000 nm in diameter. Tumor vesicles (large oncosomes) are produced by releasing tumor cells and are about 1-10 μm in diameter. The apoptotic body/bleb is about 50-2000 nm in diameter and is produced by apoptosis. Recently, a type of EVs called migrasomes has been discovered 20-23. During migration, cells produce many retraction fibrils along their paths, and at the tips and intersections of these retraction fibrils, small vesicles of about 0.5 μm-3 μm diameter are produced. Since the formation of these vesicles depends on cell migration, they are called "migrasomes". Migrasomes contain small vesicles of 50 to 100 nm diameter and are also called pomegranate-like structures because they resemble pomegranates.

Additionally, there are numerous understudied subtypes of EVs. Due to the differences in the size of different EV subpopulations and the biomolecules they contain, some groups are characterizing the composition of EV subpopulations. Several studies have classified EVs into additional subpopulations based on surface proteomic analysis or transcriptional profiles of individual EV populations 24. While there are numerous methods for isolating exosomes, none of them completely purifies specific subpopulations. The isolated subpopulations are usually enriched in a subpopulation with other EV subpopulations.

#### Composition of exosomes

Exosomes are composed of membranes and substrates. Essentially, exosomes are derived from cell membranes, so the phospholipid bilayer structure is not much different from cell membranes. There are proteins on the membrane, such as the typical exosomal membrane proteins CD9, CD63, and CD81, which can be used as exosomes to distinguish them from other EVs 25. The other type is specific membrane proteins, unique to exosomes secreted by particular cells. For example, A33 is derived from colon epithelial cells 26, and MHC-II and CD86 are from antigen-presenting cells 27.

Exosomes contain major functional effector substrates, including proteins, mRNAs, miRNAs, noncoding RNAs, and metabolites. They can regulate the levels of RNA and proteins in cells, affecting the shape and function of cells. The majority of current research into the function of exosomes is concentrated on these aspects. Many studies have improved the efficacy of exosomes by genetically altering the contents of exosomes 28, 29.

Additionally, there is a controversy surrounding whether exosomes contain DNA. Until 2019, two studies explored this important question in depth. Robert J. Coffey et al. detected DNA from purified small EVs and non-vesicular compartments (NV) and observed that dsDNA was enriched in NV fractions but no DNA in small EVs 30. At the end of the same year, Anil K. Sood et al. reported the presence of genomic DNA (gDNA) and nuclear proteins within exosomes 31. Moreover, gDNA is mainly derived from exosomes of tumor cells rather than healthy cells. However, gDNA is primarily located in the nucleus, and the generation of exosomes is usually not associated with the nucleus. They demonstrated that tetraspanins, such as CD63, encapsulate disintegrated micronuclei, which bind to gDNA and nucleoproteins in complexes, and are finally loaded into exosomes. These studies illustrate the heterogeneity of exosomes from different sources. Exosomes containing gDNA may be biomarkers for identifying specific tumor cells.

#### Function of exosomes

Exosomes play a significant role in intracellular communications 14, 32. For the most part, the biological features of exosomes reflect themselves in three ways, including that exosomes can carry and transmit biological information. Firstly, exosomes transport nucleic acids, lipids, proteins, and other signaling molecules to regulate intercellular communications. It initially evolved during the early stages of evolution and potentially affected the behavior of target cells in several ways 33.

Secondly, exosome components, such as mRNAs, lipids, and microRNAs, can influence protein modification and placement, thereby regulating the receptor cell phenotype and function. On the other hand, exosomes play a crucial role in transferring and removing components from cells. Furthermore, exosomes have a role in immunoregulation. Exosomes have been found to reduce the proliferation and differentiation of T cells *in vitro*, as well as suppress IFN-γ production from T cells 34.

Additionally, a recent study found an important role for exosomes in regulating cell size. They demonstrated that 3D cell culture reduced the size of MSCs by reducing cytoskeletal tension to increase EVs and exosome excretion 35. It gives a deeper understanding of the role of exosomes in maintaining cell size homeostasis and solves the clinical translation problem of capillary blockage caused by excessive stem cell volume.

### Sources of exosomes

Exosomes released by specific tissues and cells have distinct components and unique features. Currently, stem cells are the most concerned source of exosomes. Exosomes of skin stem cell populations are equally attractive, but research is in its infancy. Immune cells and endothelial cells have also been of interest. Furthermore, induced pluripotent stem cells (iPSCs) are the most promising source of engineered exosomes. This section will systematically discuss the features and functions of exosomes, which may contribute to wound healing, from various sources.

#### Human umbilical cord mesenchymal stem cells

The most widely employed stem cells in clinical studies are human umbilical cord mesenchymal stem cells (hucMSCs), multipotent and self-renewing stem cells. Under particular conditions, hucMSC can differentiate into the cell types that comprise human tissues and organs 36. The hucMSCs have a wide range of clinical applications since they express all of the fundamental properties of bone marrow MSCs and demonstrate high proliferation, differentiation potential, and minimal immunogenicity 37. These advantages make hucMSCs the most promising stem cell-based drug delivery system. Additionally, multiple studies have been conducted to determine the efficacy of hucMSC therapies for various diseases, and critical therapeutic advancements have been accomplished with these cells 36, 38, 39. Furthermore, hucMSC-derived exosomes (hucMSC-exos) have demonstrated similar efficacy to hucMSCs in various diseases. For example, anti-apoptotic, anti-inflammatory, and promoting tissue regeneration 40-43. However, the mechanisms behind these benefits are unclear, and most research was conducted on animal models. The utility of hucMSC-exos in human clinical trials should be investigated further.

#### Adipose-derived stem cells

Adipose-derived stem cells (ADSCs) are isolated from adipose tissue that exhibits multilineage differentiation, high proliferation capability, and self-renewal properties 44, 45. Since adults have lost the opportunity to store the umbilical cord and placenta, obtaining stem cells from adipose tissue is an alternative option. ADSC-derived exosomes (ADSC-exos) are key paracrine components secreted by ADSCs, having a variety of biological functions. ADSC-exos have garnered considerable interest due to their role as paracrine mediators, contributing to tissue regeneration. ADSC-exos have been demonstrated to promote cutaneous wound healing by regulating the HDFs, HaCaTs, and other major target cells via various signaling pathways 46, 47. Furthermore, ADSC-exos may enhance collagen synthesis during cutaneous wound healing by upregulating the PI3K/Akt pathway 48. According to Li X. et al. 49, exosomes from NF-E2-related factor 2 (Nrf2)-overexpressing ADSCs dramatically decreased the ulcerated area in the foot of diabetic rats by angiogenesis and boosted endothelial cell proliferation to promote wound healing.

#### Bone marrow-derived mesenchymal stem cells

Bone marrow mesenchymal stem cells (BMMSCs) are one of the most widely used stem cells in clinical research. Compared with hucMSCs and ADSCs, BMMSCs are more challenging to isolate and riskier to obtain. However, their effects for different indications are not the same and cannot completely replace each other 50, 51. In experimental models of diverse pathologies, the utilization of BMMSCs-derived exosomes (BMMSC-exos) as a possible approach for future therapeutics has been investigated. These studies have confirmed that exosomes can improve tissue fibrosis and promote tissue regeneration through anti-apoptosis, anti-oxidation, and anti-inflammation 52-55. Since these exosomes are derived from bone marrow, previous studies usually focus on bone regeneration and internal organ repair. Recently, studies have confirmed that BMMSC-exos can also promote wound healing. BMMSC-exos promote the proliferation and migration of human spontaneously immortalized keratinocyte cell line (HaCaT) through the miR-93-3p/apoptotic peptidase activating factor 1 (APAF1) pathway 56. Although the effect of BMMSC-exos in promoting skin-related cell proliferation was good *in vitro*, the results of *in vivo* studies suggested that BMMSC-exos had a limited impact on chronic wounds 57, 58. Therefore, further improvement of exosomes is needed to improve the targeting of indications.

Furthermore, a study showed an interesting result: the beneficial effects of BMMSC-exos in healthy rats were better than in diabetic rats 59. It suggests that exosomes derived from various cell types have different therapeutic properties (52), and exosomes derived from unhealthy individuals may have different substrates. Therefore, it is necessary to ensure a strict quality control system when treating with stem cells or exosomes.

#### Other mesenchymal stem cells

Mesenchymal stem cells (MSCs) are pluripotent stem cells and vital members of the stem cell family. Unlike other stem cells that are derived from specific tissues, MSCs are a general term for a class of stem cells that can come from a variety of different tissues, including adipose tissue, muscle, liver, placenta, amniotic fluid, blood, pulp, brain, bone marrow, spleen, kidney, thymus lung, and pancreas 60-62. For example, the hucMSCs, ADSCs, and BMSCs described above are all MSCs. MSCs exhibit various characteristics that distinguish them from other cell types, making them an ideal source of exosomes.

Currently, hucMSCs and BMSCs are the most registered MSCs in clinical studies 63, 64, and autologous ADSCs are widely used in cosmetic surgery. In terms of technical maturity and usage safety, they are more likely to be used as sources of exosomes in future applications. This is the reason why we focus on these three MSCs. Besides, many other MSCs are effective in wound healing, but relatively few studies have been performed. For example, umbilical cord blood MSCs and their exosomes inhibited TGF-β receptors through the high expression of miR-21-5p and miR-125b-5p to promote traumatic tissue regeneration and reduce scar formation 65. Human amniotic fluid MSCs and their exosomes suppressed TGF-β expression by upregulating miRNAs (let-7-5p, miR-22-3p, miR-27a-3p, miR-21-5p, and miR-23a-3p) to promote wound healing and inhibit scar formation 66. Another study showed that both placental MSCs and their exosomes promoted wound healing and skin appendage regeneration with significantly better results than ADSC-exos 67.

Furthermore, preliminary findings suggest that some immunomodulatory features of MSCs can be transferred to their exosomes, enhancing the lifespan of the MSC exosome-derived drug delivery vehicle and improving drug bioavailability. Additionally, when the exosome-associated protein CD81 was quantified to determine the number of exosomes secreted by different cell types, MSCs produced the highest amount of exosomes 68. On the contrary, while MSCs have a high capacity for expansion *in vitro*, this does not bode well for the long-term viability and large-scale synthesis of MSC exosomes. However, prior studies have shown that using Myc oncogene immortalized MSCs can overcome this feature 69. Immortality reduces the MSCs differentiating capability without impairing exosome synthesis or therapeutic efficacy. All in all, MSCs are an excellent source of exosomes due to their immunomodulatory features and prolific synthesis of the exosome.

#### Skin stem cell populations

Indeed, the skin has a strong self-healing ability, attributed to its rich population of stem cells, such as epidermal stem cells (ESCs) and hair follicle stem cells (HFSCs). Both have been shown to have significant clinical applications; however, research on them is still in its initial stages. In addition to these two stem cell populations, skin also contains many other stem cell populations. However, many studies are not mature due to the limitation of the technology. Therefore, this section focuses on the discussion of ESCs and HFSCs.

ESCs are critical cells that maintain epidermal renewal and repair. After wounding, ESCs near the traumatic margin migrate to the wound surface to promote re-epithelialization. Recent studies have shown that ESCs and their exosomes can promote wound healing. For example, ESCs exosomes inhibit the activity of TGF-β1 and its downstream genes by upregulating the substrates miR-16, let-7a, miR-425-5p, and miR-142-3p to promote traumatic tissue regeneration as well as reduce scarring in rats 70. Additionally, Ogawa et al. found that human immortalized epidermal cell line (HaCaT) pretreated with Fisetin can secrete exosomes to activate HFSCs proliferation 71.

Similarly, HFSCs have multidirectional differentiation potential and are one of the important stem cells for maintaining skin renewal and repair 72-74. Under normal conditions, HFSCs are present only in hair follicles and are not involved in wound repair 72, 75-77. However, after stimulation by wounding, HFSCs will migrate to the wound surface and differentiate into epidermal cells to promote re-epithelialization. Studies have proven that hair follicle grafting can promote the regeneration of traumatic tissue 78. *In vitro* culture of HFSCs has been challenging, but recently this issue has been solved 79. These studies provide the basis for the future use of artificial HFSCs and their exosomes for wound treatment. Additionally, dermal papilla cells-derived exosomes have been shown to promote hair follicle growth 80-82. Although these studies did not clearly state that this exosome promotes wound repair, it can be speculated that it may also have a cooperative function in promoting skin cell regeneration.

#### Macrophages

Inflammation is an inevitable stage of cutaneous wound healing, inseparable from the regulation of macrophages. Injured tissues secrete various cytokines and chemokines to recruit monocytes, which will be polarized into different subtypes in the injury microenvironment. The most common phenotypes are the pro-inflammatory M1 and anti-inflammatory M2 subtypes 83-85. Typically, macrophages are first polarized to the M1 subtype, and their main function is to clear pathogens and dead cells. In the next stage, the macrophages gradually transform into the M2 subtype, and their main functions include suppressing inflammation and promoting angiogenesis and tissue repair. In the cutaneous wound healing process, the ratio of M1/M2 is critical. Excessive activation of M1 exacerbates tissue damage, and inhibition of M2 activation reduces healing capacity 86.

Furthermore, a study based on M2 exosomes showed that subcutaneous injection of M2 exosomes into the wound edge reduced the M1/M2 ratio by promoting macrophage reprogramming, thereby enhancing angiogenesis, re-epithelialization and collagen deposition to accelerate wound healing 87. Currently, macrophage-based medical devices available on the market are used to treat wounds. Therefore, we think macrophage-derived exosomes are also potential drugs for wound treatment.

#### Endothelial progenitor cells

Endothelial progenitor cells (EPCs) are a heterogeneous population of mononuclear cells capable of migrating and differentiating in wound sites or performing paracrine functions. Exosomes have acquired significant attention among the EPCs' secretomes due to their potential for cell-free biological treatment, biocompatibility, and immunogenic properties. As a result, EPC-derived exosomes (EPC-exos) are attractive candidates for cell-free therapies and novel drug delivery systems in treating a variety of pathologies, including cardiovascular diseases, bone diseases, brain damage, and diabetic complications. Numerous studies have demonstrated that EPC-exos reduce hypertension, dyslipidemia, circulating cytokine levels, and arterial remodeling by modulating immune cells and oxidative stress levels 51, 88-91. Importantly, EPC-exos can avoid the risk of a pulmonary embolism due to oversize EPCs volume while reducing inflammation and tissue damage 51, 92. Besides, EPC-exos exhibits increased stability, biocompatibility, and immunogenicity. EPC-exos also showed excellent efficacy in promoting wound healing. Several studies have confirmed that EPC-exos promote angiogenesis by up-regulating angiogenesis-related factors and regulating ERK1/2 and miRNA-221-3p to accelerate the healing of chronic diabetic wounds 93-96. As a result, EPC-EVs may offer promising prospects for innovative cell-free therapeutics in treating various diseases.

#### Human induced pluripotent stem cell

**Figure 2:**
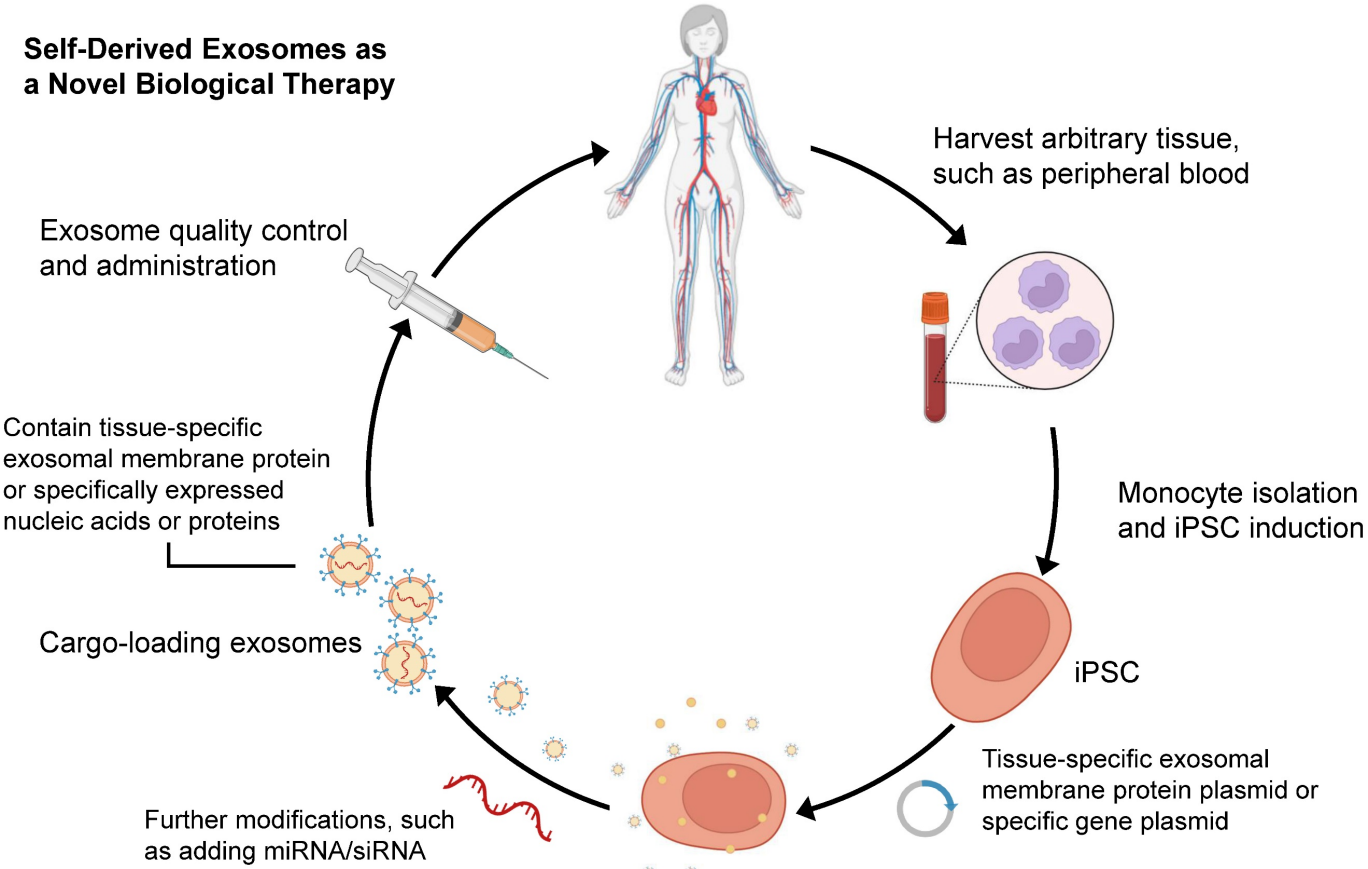
** Schematic process for producing self-derived exosomes as a new biological theranostic agent. Generally, any somatic cell can be induced to produce iPSCs for exosome production by *in vitro* reprogramming.** For example, peripheral blood mononuclear cells can be induced into iPSCs, and then plasmids containing specific genes can be introduced into the cells. Exosomes can be harvested from cell culture and modified to increase their substrates and functions. Particularly, quality-controlled exosomes can well meet the requirements of clinical application.

Since Yamanaka et al. demonstrated that retrovirus systems could deliver Oct3/4, Sox2, c-Myc, and Klf4 into somatic fibroblasts. Fibroblasts could be transformed into pluripotent stem cells and are hence referred to as induced pluripotent stem cells-derived exosomes (iPSCs) 97. The iPSCs can develop into any cell lineage in the body and have regenerative characteristics 98. Recently, iPSC-derived exosomes (iPSC-exos) have been shown to have therapeutic benefits, mainly through a paracrine pathway 99. Numerous studies have successfully demonstrated the efficacy of iPSC-exos in wound healing. Zhang et al. established the first use case of human iPSC-derived MSCs (hiPSC-MSCs) in treating rat cutaneous wounds 100. Subcutaneous injections of hiPSC-MSC-exos around rat wound sites led to rapid re-epithelialization, decreased scar widths, and collagen maturation. These findings indicate that hiPSC-MSC-exos can stimulate collagen production and angiogenesis, thereby accelerating the healing of cutaneous wounds. Additionally, Kobayashi et al. reported that diabetic ulcer mice treated with exosomes from undifferentiated hiPSCs showed quicker wound closure and healing rates 101. In an *in vitro* scratch assay, these exosomes promoted fibroblast migration and proliferation. To assess the potential of iPSC-exos in human clinical trials, Lu et al. treated the wounds with exosomes derived from allogeneic rhesus macaque iPSCs 102. They observed rapid epithelialization, collagen deposition, and angiogenesis in skin wounds. In addition to customized treatment employing autologous exosomes, iPSC-exos might be a potential alternative for disease therapy (figure 2). Despite these encouraging advances, the possible use of allogeneic iPSC-exos in different disease models requires more investigation and evidence.

#### Others

The main source of exosomes in the laboratory is the supernatant of the cell culture medium. Two main sources of such supernatants are culturing cells in fetal bovine serum (FBS) medium depleted of extracellular vesicles and culturing cells in an FBS-free medium. However, it remains unclear which is superior and its effect on cells. Although it is still inconclusive, the current mainstream method is more inclined to the former.

Clinical products have extremely high requirements on the source of raw materials. Many biological products of animal origin are generally unable to obtain clinical use licenses. These animal-derived products are likely to contain some potentially pathogenic microorganisms, contaminants, and allergens. Therefore, the serum-free medium is an industrial direction that has been vigorously developed in recent years. There are a variety of commercial serum-free medium products have been developed. However, different types of cells usually have varying requirements for the medium. More targeted development is required if special cell-derived exosomes are to be studied.

The above descriptions are all important sources of exosomes related to wound healing. In addition, there are many other sources of exosomes. These exosomes often exhibit a strong ability to promote cell proliferation and may also be a potential therapeutic agent.

### Methods of exosome isolation

Centrifuging, a heterogeneous mixture, separates the particles according to their shape, size, and density. Denser or bigger particles settle first 37. Preparative ultracentrifugation is classified into differential ultracentrifugation and density gradient ultracentrifugation. Exosome separation by ultracentrifugation typically involves several centrifugation cycles with varied force and duration to isolate exosomes in a sample based on size and density. Ultrafiltration utilizes an ultrafine Nano-membrane with a distinct molecular weight cut-off (MWCO) to extract extracellular vesicles from cell culture medium or clinical samples and separate exosomes from co-vesicles based on their size 39. Compared to ultracentrifugation, exosome isolation through ultrafiltration significantly reduces processing time and does not require specialized equipment, making it an excellent alternative to conventional ultracentrifugation 40. Notably, ultrafiltration enables researchers to select specified subsets of tiny extracellular vesicles (including exosomes) with predetermined particle sizes by readily altering the filter size 41.

Size-exclusion chromatography (SEC) was developed by Grant H.L. and Colin R.R. in 1955 103. A liquid sample is subjected to several fates after passing through a porous stationary phase. Larger molecules unable to pass through the stationary phase pores are trapped and released later. SEC is widely utilized for the high-resolution separation of large molecules such as polymers, proteins, and liposome particles 49, 50. Both exosomes and liposomes share many physical features. The information gained through SEC-based liposome isolation may easily be used for exosome separation. Nowadays, companies have introduced commercial SEC kits built exclusively for exosome isolation in a short time. The revelation that certain proteins and receptors are expressed in all exosomes irrespective of origin enabled the development of immunoaffinity-based exosome extraction based on the binding specificity of such protein markers and their corresponding antibodies 55. Theoretically, any protein or component of the cell membrane that is exclusively or predominantly present on the membrane of exosomes and does not have soluble equivalents in extracellular fluids might be employed to collect exosomes by immunoaffinity. Over the last few decades, numerous exosome markers have been identified, including heat shock, transmembrane, lipid-related, and fusion proteins 56, 57. For selective exosome isolation, various transmembrane proteins have been widely explored 58, 59, enabling the development of popular exosome isolation kits, including Exosome-human CD63 isolation reagent (Thermofisher) and Abcam Exosome isolation and analysis kit 104.

### Ideal method for exosome isolation

**Figure 3:**
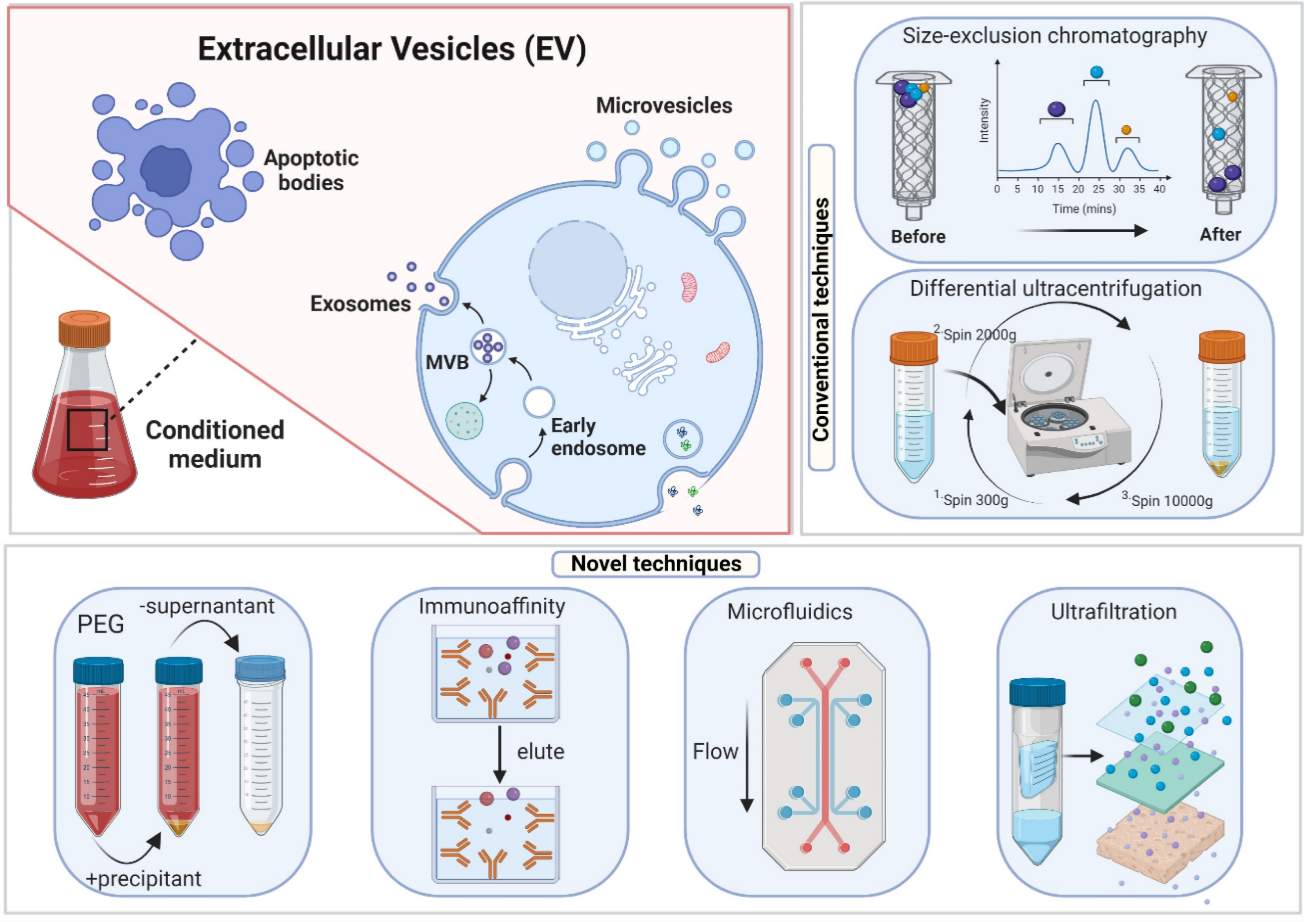
Extracellular vesicle (EV) biogenesis, subpopulations, and conventional and novel methods of exosome isolation.

An examination of the exosome literature published before 2015 showed that 81% of research utilized ultracentrifugation as an isolation method 105, 106. However, between 2014 and 2017, the use and popularity of this traditional technique declined, most likely due to technological breakthroughs in exosome extraction that need less time and work (figure 3). Given the various isolation approaches stated above, each with advantages and limitations, EV researchers have not agreed on an optimum strategy. None of these strategies are exclusively optimum for exosome isolation. Hence there is an understanding that an inclusive approach employing several techniques may generate the most significant results.

The Poly-Ethylene Glycol (PEG)-Based Precipitation approach wraps exosomes in aqueous PEG 68, which facilitates the development of exosome aggregates. Due to the co-precipitation of non-exosomal soluble proteins, purity and specificity are lost. The final pellet from a PEG-based exosome extraction technique contains non-exosomal proteins, immunoglobulins, virus particles, immunological complexes, and other contaminants 26, 107. Immunoprecipitating exosomes using exosome-specific markers such as CD9 or other tetraspanins from a PEG-based pellet can circumvent the lack of purity in the exosomal preparations but leads to "biased" isolation 107, e.g., isolating the CD9+ exosomal population while excluding the CD9- exosomes. Due to its non-specific mechanism, this approach isolates high-yield but low-quality exosomes. Combined with an immunoprecipitation technique, it can generate pure exosomal fractions.

Recently, novel approaches have been used with various exosome extraction techniques to enhance the purity of exosomes 108, 109. Dual-mode chromatography (DMC) was effectively employed to minimize the contamination of plasma exosome preparations with lipoprotein particles (LPPs) 109. This method combines two separation processes: removing high-density lipoproteins (HDLs) by SEC and using cation exchange to separate positively charged LPPs from negatively charged exosomes. Another approach, Flu-SEC or F-SEC, combines SEC with fluorescence detection. SEC can be combined with detecting fluorescently-labeled exosomes to maximize exosome isolation using high-performance liquid chromatography and a fluorescence detector 108. Further hybrid strategies that utilize both SEC with PEG and differential ultracentrifugation (dUC) can also be used. It is essential to remember that outside of EV research, SEC is a typical procedure for the purification and fractionation of peptides due to its excellent reproducibility and stability 110.

Methods for isolating exosomes are not standardized in terms of methodology. This gap has prompted the development of innovative strategies to enhance exosome extraction from various body fluids 111-113. Currently, the ideal EV separation technique is determined by the quantity and nature of the starting material, the availability of specialized equipment, the planned therapeutic usage, the route of administration, and the desired final result. Considering the variety of exosome sources, developing an optimized technique for isolating exosomes from various samples would be advantageous. Researchers must balance the separated vesicles' purity, efficiency, and downstream uses. A combination methodology developed by thoroughly examining dUC, UF, PEG-based precipitation, immunoaffinity capture, microfluidics, and SEC techniques would be ideal. In addition to being unable to differentiate between exosomes and microvesicles of the same size, SEC-based exosome isolation techniques must avoid denaturation of the biological targets and control for unwanted electrostatic and hydrophobic interactions between the mobile phase containing the vesicles and the stationary, porous phase. Based on efficacy, reliability, repeatability, and usability, an SEC-coupled method for isolating exosomes with a high yield of homogeneous, intact exosomes seems ideal.

#### Strategy to increase the yield of exosomes

The conventional extraction methods usually yield only small quantities of exosomes. Such a low yield has impeded the growth of fundamental research into exosome analysis and its diverse applications. To combat this, several attempts have been undertaken to investigate upstream and downstream alterations to boost exosome production.

The approaches now available for the extraction of exosomes are mostly based on their chemical, physical, and immunological features and have been modified from those used for extracting viruses and microvesicles. To present, only a handful of technologies and facilities were built specifically for exosome extraction 107, 114, 115. However, modifications in exosome extraction procedures may affect not only exosome yield but also their size, structure, and biofunction 116. Therefore, isolation methods must be carefully chosen depending on the objective of the research as well as the method and sample characteristics (table 1). Notably, with the integration of many approaches in a progressive way, the overall production of exosomes could be substantially enhanced. Similarly, strategies to increase the yield of exosomes can be developed by integrating various existing techniques. For example, using 3D microcarriers based suspension cultures may enhance the output of exosomes when specific soluble factors and/or bioactive microcarrier materials are introduced 117, 118. In addition, whole or semi-artificial exosomes may be evaluated for mass manufacturing due to their relatively controlled composition and well-defined therapeutic pathways 119, 120. Semi-artificial exosomes may enhance the function and yield of exosomes produced from stem cells. By co-incubating extracellular vesicles with drug-loaded liposomes (with or without PEG), it was possible to make a hybrid exosome with great cargo capacity, absorbability, and ability to target. This may ultimately result in improved therapeutic outcomes 114, 121.

Liposomal stimulation may be an effective method for increasing exosome production. However, further preparation to improve exosome purity may be required. The liposome, an artificial vesicle consisting of lipids and cholesterol, is biocompatible and has a high drug-loading capability. Despite this, liposomes have limited clinical applications due to their undesirable immunogenicity and unsatisfactory stability, biocompatibility, targeting ability, and absorption 122, 123. Integrating the benefits of exosomes and liposomes can mitigate these downsides substantially 124, 125. In addition, it is possible to fine-tune the induced exosome's biological features by manipulating the stimulating liposomes' physicochemical parameters. In the future, by combining various portfolios of the aforementioned strategies, it could be possible to identify the ideal method for extracting desired quantity and quality of exosomes.

### Mechanism to regulate exosomes function during cutaneous wound healing

When an injury occurs, not only does the skin's barrier function becomes compromised, but also sensitive to temperature, pain, and touch. Wound healing is a dynamic biological process commonly classified into four phases: inflammation, angiogenesis, proliferation, and remodeling 133, 134. In these respects, multi-cell type growth factors, enzymes, cytokines, and structural matrix proteins coordinate and control these processes 135. Damage to the endothelium, exposure to the basement membrane, and subsequent blood component spillage are consequences of the initial injury. When the injury occurs, vasoconstriction is the initial response to the damage caused by prostaglandin and thromboxane production.

Meanwhile, platelets attach to the exposed collagen and release their granule contents, while tissue factors stimulate platelets and coagulation cascades 136, 137. Collagen, platelets, thrombin, and fibronectin-derived blood clots control haemorrhages and protect the wound by providing matrix and soluble components that promote adhesion 138. Besides, dermal fibroblasts are a critical cell type in the normal wound-healing process 55. The primary task of dermal fibroblast is the production of extracellular matrix (ECM), wound contraction, collagen synthesis, re-epithelialization, and tissue remodeling. The disruption and prolongation of the wound-healing process can cause scarring and poor healing quality 55. It results in functional impairment and can negatively impact the patient's mental health. Therefore, minimizing healing time and scar formation after an injury is a crucial therapeutic need.

**Figure 4:**
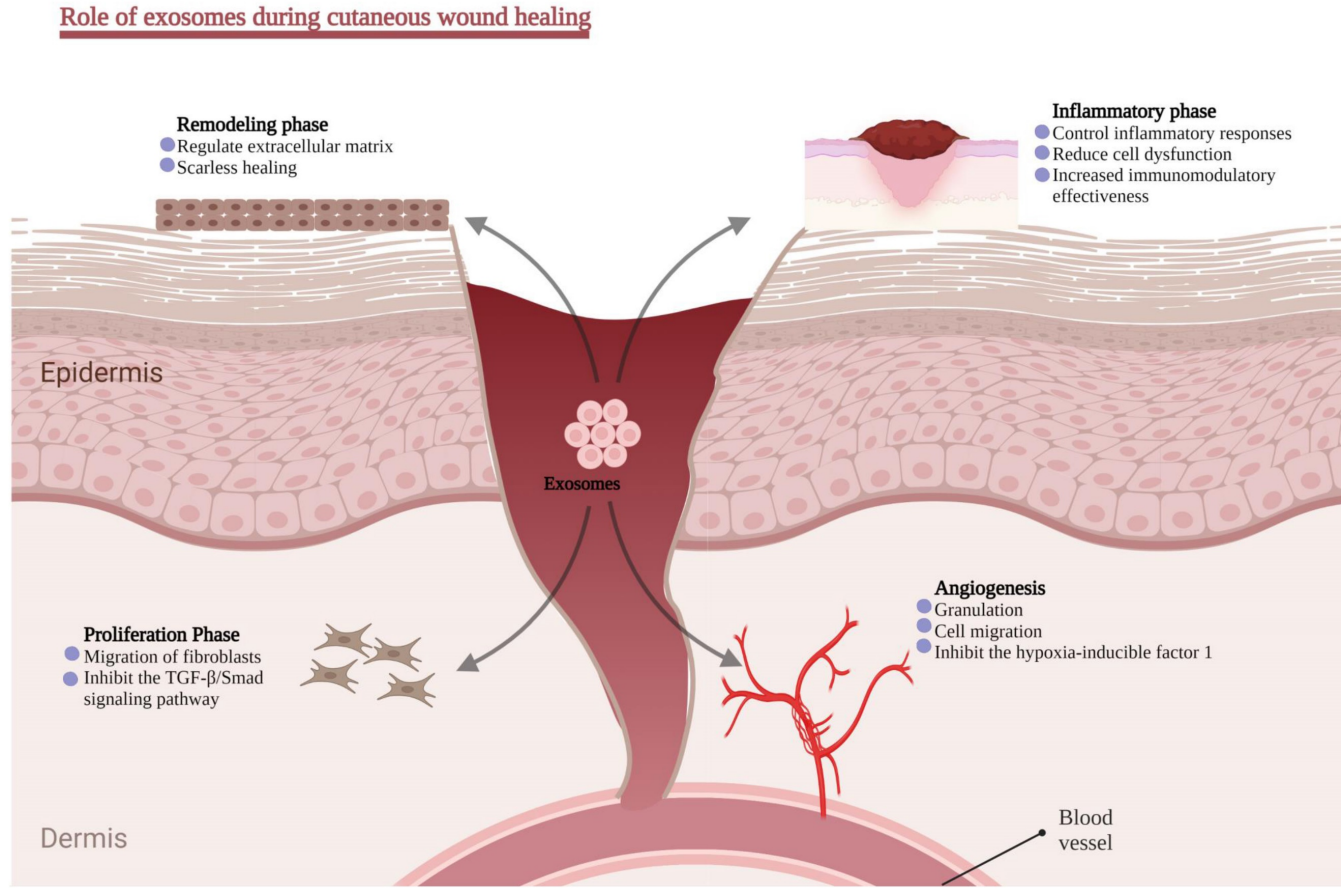
Role of exosomes in different stages of wound healing.

Cutaneous scar formation results from the poor wound-healing process and involves a coordinated sequence of interactions involving cells, ECM components, and signaling molecules. Scar tissues exhibit typical characteristics of excessive ECM deposition and the absence of epidermal appendages such as sweat glands and hair follicles. ECM remodeling, specifically collagen synthesis and degradation, is the major molecular and cellular event contributing to scarring. Meanwhile, the transition of fibroblast into myofibroblast is also crucial. Numerous studies have demonstrated that the TGF-1/Smad signaling pathway is implicated in collagen production and fibroblast transformation into myofibroblast 139. This section will briefly introduce the regulatory role of exosomes in ordinary wound healing (Figure 4).

#### Inflammatory phase

Inflammation is the first of four wound-healing responses and begins within 24-48 hours after an ischemic. During the early stages of wound healing, hyperemia, serous exudation, leukocyte infiltration, and local redness and swelling are the early inflammation signs. Generally, a mild inflammatory response is helpful because it helps eliminate inflammatory factors, fight off infections, and clear cell debris, all of which aid in regenerating injured tissue. In contrast, a hostile inflammatory environment plays a significant role in developing chronic wounds 140.

Vasodilation and increased capillary permeability cause edema during the inflammatory phase. Immune cells produced from bone marrow mediate essential processes in wound healing by removing pathogens, apoptotic cells, and cell debris 141. Subsequently, neutrophils decompose waste and wounded tissues by secreting proteases and protecting the wound from microbial and pathogenic infections through oxygen-dependent processes. Meanwhile, local monocyte phagocytosis of apoptotic cells and cell debris is essential in maintaining tissue homeostasis 142. Interestingly, CD4+ T cells have been found to promote wound healing, while CD8-T cells have a detrimental effect on wound healing 143. Inflammation eventually increases the macrophage transition from M1 to M2 144.

MSCs act as immunomodulatory factors and control inflammatory responses by releasing exosomes, thereby reducing the adverse effect of immune cell dysfunction on wound healing. The regulation of the inflammatory response is inextricably linked to exosomes containing a diverse array of proteins and RNAs. Recent research indicates that miR-223 is substantially expressed in exosomes derived from MSCs. By inhibiting the lipopolysaccharide-induced TLR4 signaling pathway, the overexpression of miR-181c by hucMSC-exos reduces inflammation in a burn-induced rat model. The study revealed that exosome-derived miR-181c modulates inflammation 37. It is worth noting that some substances can remarkably improve the paracrine impact of MSCs. Recently a study found that LPS pre-conditioned exosomes had a superior capability to reduce inflammation than unconditioned exosomes. It might be because let7b/TLR4 pathway is linked to macrophage polarization 145. Additionally, pretreatment of hucMSCs with interleukin-1 beta (IL-1β) increased the immunomodulatory effectiveness, which was mediated in part via exosome-driven miR-146a transfer (a well-known anti-inflammatory microRNA) 146. These findings paved the way for new research avenues and implicational opportunities for exosomes in this domain of abnormal wound healing caused by inflammatory regulatory function.

#### Angiogenesis

Angiogenesis is a crucial phase in the healing process. Granulation tissue is structured or encapsulated with necrosis, thrombosis, and inflammatory exudates to facilitate keratinocyte migration, generating a new matrix that allows the migration of keratinocytes later in the healing process. An adequate blood flow toward the wound site offers nutrition, oxygen, and cell migration paths necessary for proper tissue regeneration.

Numerous studies have revealed the angiogenesis-promoting potential of stem cell-derived exosomes 147. For instance, multiple exosome-related investigations showed that conserved ADSC-secretome proteins are identified in a differentiating or pro-inflammatory stimulus 148, 149. Cell stimulation regulates the protein content of exosomes compared to parental cells. ADSC-exos may successfully decrease the ulcerated regions and enhance endothelial progenitor cell proliferation and angiogenesis in a high glucose environment, especially when ADSC-exos overexpress the transcription factor Nrf2 49. A further study discovered that ADSC-released MVs include numerous microRNAs implicated in the proangiogenic action and target miR-31 in human umbilical vein endothelial cells (HUVECs). This work demonstrated the potential for stem cell-derived MVs in proangiogenic treatment 150. Liang et al. found that MiR-125a was abundantly present in the ADSC-exos, which may transport miR-125a to endothelial cells, suppress the angiogenic inhibitor delta-like 4 (DLL4) expression, and alter endothelial cell angiogenesis by encouraging the development of endothelial tip cells 151. These findings suggest that stem cell-derived exosomes might be a priority option for wound angiogenesis, but their protein composition needs further exploration to elucidate their angiogenesis capabilities.

#### Proliferation phase

Re-epithelization begins during the proliferative phase. The dermis relies on epithelial cells migrating from the wound edges and any surviving adnexal structures. This phase is often referred to as the development of new tissue. Additionally, epithelial cells began migrating from the edge to the wounded region to cover the wound surface, and then matrix proteins offered scaffold structures for cell attachment and healing. In order to repair the epithelial barrier, epithelial cells continue to migrate and proliferate. M2 macrophages play a role in the migration and growth of keratinocytes and endothelial cells during tissue regeneration 152. Fibroblasts start secreting a great deal of immature type III collagen into the matrix 153. Similar to the essential role of miRNA in promoting angiogenesis, they also play a crucial role in the proliferation of fibroblasts. Choi et al. discovered that treating human dermal fibroblasts with ADSCs-exos enhanced the miRNA expression inside the fibroblasts and contributed to healing 154. Simultaneously, miRNA chip analysis revealed that the expression of 292 distinct miRNAs was altered during the proliferation and differentiation of dermal fibroblasts, with 199 being up-regulated and 93 being down-regulated.

#### Remodeling phase

During the remodeling stage, the fibroblast continues to produce collagen 155, 156, and the level of collagen type III declines with time and is gradually replaced by collagen type I 157, 158. A recent study found the MSC-exos dose-dependent effect on the migration and proliferation of normal and diabetic chronic wound fibroblasts. However, MSC-conditioned media-deficient exosomes did not impact the fibroblasts 148. Hu et al. studied the effect of exosomes generated by ADSCs on skin wound healing and fibroblast activity using a rat model. *In vitro* tracking tests demonstrated that fibroblasts may internalize tagged ADSC-exos. Simultaneously, immunohistochemistry, Masson staining, and qRT-PCR analysis revealed ADSC-exos-stimulated Collagen I, PCNA, N-cadherin, cyclin-1, and III gene expression. Besides, the elastin protein synthesis is dramatically elevated in a dose-dependent manner, indicating that exosomes enhance wound healing in the early phases.

Exosomes may hinder collagen synthesis in the later stages of wound healing. Interestingly, intravenous injection appears to have a much quicker and better impact on wound healing than local injection. Inguinal wounds (with a fat layer) healed more quickly and had a higher concentration of exosomes in the area surrounding the wound location than dorsal wounds (without a fat layer) 159. Which component of exosomes contributes to this event? Recent advances indicate that ADSC-exos can induce HDF cell migration and angiogenesis during wound healing. It primarily depends on metastasis-associated lung adenocarcinoma transcript 1 (MALAT1), a lncRNA that typically works in the pre-RNA nucleus, splicing, and angiogenesis 160, 161. Therefore, exosomal lncRNAs contribute significantly to their therapeutic potential, including tissue repair and regeneration.

Extracellular matrix remodeling might take two weeks to over a year to complete. The formation and remodeling of the extracellular matrix play a significant role in determining the degree of scarring throughout the remodeling phase of wound healing. In the later stages of wound healing, the apoptosis effector cells, the degradation of ECM by matrix metalloproteases, the replacement of collagen III with collagen I, and the production of additional ECM proteins have all been documented in the preclinical studies 162. Pelizzo, G. (2018) investigated the effects of intradermal injections of ADSC-EVs and BMMSC-EVs in a cutaneous wound healing model in an experimental setting. After ADSC-exos inoculation, it was possible to acquire well-regenerated tissue with a complete epithelial layer, dermal papillae, cutaneous annexes, and repaired connective matrix 163.

Interestingly, scarless healing transpires in the early embryonic development or midgestation phases. Thus, the ratio of collagen type III to type I and TGF-β3 to TGF-β1 is higher in fetal wound tissue. *In vivo*, ADSC-exos raised the ratios of collagen III to collagen I as well as TGF-β3 to TGF-β1, decreased fibroblast differentiation into myofibroblasts, and inhibited granulation tissue development compared to the control group. Exosomes also stimulated the ERK/MAPK pathway and raised the ratio of MMP3 to TIMP1 in dermal skin fibroblasts, enabling scarless cutaneous healing through regulating ECM remodeling 139.

### Application of exosomes in wound healing

Exosomes' most attractive point is replacing stem cells with a new generation of biological treatment methods. Since the clinical application of stem cell therapy has just begun, there are no clinical trials of exosomes in wound healing. Existing exosome applications are mainly based on animal studies. These studies are based not only on mechanical injury wounds but also on injuries caused by many special factors, such as burns, diabetic ulcers, and radiation. The healing process for these injuries is more complex than for mechanical injuries. In this part, we will focus on the research progress of exosomes in non-mechanical injury.

#### Burns

Burns damage the skin and internal organs caused by thermodynamic changes, chemicals, and electricity. Patients with extensive burns are prone to infection and sepsis, and the key to treatment is to accelerate wound closure to reduce exposure to the environment 164. Since stem cells significantly promote wound healing, exosomes' role in burn wound healing has also attracted attention 165, 166. Previous studies have confirmed that exosomes can promote burn wound healing. Zhang et al. showed that hucMSC-exos promote wound healing via activating Wnt4/β-catenin signaling 43. Burn-induced excessive inflammation is one major reason the tissues are difficult to heal. Li et al. demonstrated that hucMSC-exos could reduce excessive inflammation at burn sites by modulating microRNA-181c 167. These studies have confirmed that exosomes can promote the proliferation of skin cell populations, enhance cell viability, and reduce cell damage.

Furthermore, compared to local damage caused by mechanical injury, the permeability of burns has a systemic effect on the body. RNA-sequencing-based studies have found that plasma exosome-derived microRNA profiles are altered after thermal injury, which may be an important cause of disordered skin gene expression in the early burn stage 168, 169. Protein profiling-based studies have found that burns induce significant changes in serum exosomal protein function, altering enzyme inhibitor activity, heparin-binding, coagulation, and lipid transport 170. Previous studies have demonstrated that exosomes have a systemic regulatory function 171. It suggests that by restoring mRNA levels and protein functions, exosomes may promote the normal healing function of damaged tissues.

Visceral injury is a common complication of burns and is closely related to the alteration of exosomes, and it will affect wound healing and rescue. Maintaining the normal physiological level of internal organs is also the key to wound healing. A study on vascular permeability found that serum exosomes containing S100A9 may be a key factor in promoting pulmonary microvascular hyperpermeability 172. Another study found that injection of hucMSC-exos attenuated burn-induced acute lung injury by mediating microRNA-451 173. These studies demonstrate that burn-induced exosome changes are the key to causing systemic damage, and stem cell exosomes can rescue internal organ injury. Unfortunately, there are few studies on exosomes for other visceral injuries, which may be an important direction for future burn research.

#### Radiation

Radiation is highly penetrating and can cause damage to both surface and internal tissues, including DNA damage and excessive cellular stress response. Radiation-induced damage is an important clinical problem that is difficult to solve 174-176. Previous studies have demonstrated that exosomes are also beneficial in treating radiation damage 177. For example, ionizing radiation therapy can damage adjacent tissue, leading to serious wound complications. Local injection of rat plasma-derived exosomes into skin wounds created on the back of irradiated rats can be observed that exosomes modulate cell proliferation and ferroptosis in irradiated fibroblasts, thereby promoting wound healing 178. Ultraviolet radiation causes skin damage through excessive inflammation and oxidative stress 83. The hucMSC-exos can repair ultraviolet radiation and oxidative stress-induced skin damage through adaptive regulation of the Nrf2 defence system 179. A similar study also confirmed that hucMSC-exos alleviated UV- and H_2_O_2_-induced skin damage by modulating the SIRT1 pathway by delivering 14-3-3ζ protein 180. Another ultraviolet radiation-based photoaging study revealed that exosomes derived from 3D-cultured human dermal fibroblasts have properties for the prevention and treatment of skin aging 181.

#### Diabetic ulcer

A diabetic ulcer is one of the common complications of diabetes, the incidence of which accounts for about 20% of diabetic patients 182-184. Diabetic ulcers are difficult to heal, and patients with severe ulcers even require amputation, bringing physical and economic burdens 74, 185, 186. Currently, there is a lack of effective treatment drugs, and the most effective method is nursing management. Stem cells and exosomes hold promise for diabetic ulcer treatment.

A variety of stem cell-derived exosomes has been shown to contribute to the recovery of diabetic wounds 187. For example, ADSC-exos promote angiogenesis and tissue regeneration by regulating inflammation, oxidative stress, and cytokine levels 49, 188. Platelet-rich plasma exosomes improve angiogenesis and re-epithelialization in chronic wounds by inducing the proliferation and migration of endothelial cells and fibroblasts by activating YAP signaling 189. EPC-derived exosomal microRNA-221-3p promotes wound healing 93, 96. Human circulating fibroblast exosomes promote wound healing by modulating angiogenesis, anti-inflammatory, and collagen synthesis 190. Menstrual blood-derived MSC-exos reduce scarring and improve non-healing wounds by modulating macrophage polarization 191. The iPSC-exos can accelerate wound closure 101. Persistent inflammation is also one of the reasons why diabetic ulcers are difficult to heal. Injection of RAW264.7 macrophage-derived exosomes can exert anti-inflammatory effects by inhibiting the secretion of pro-inflammatory enzymes and cytokines, thereby inducing endothelial cell proliferation and migration, promoting angiogenesis and re-epithelialization, and accelerating diabetic wound healing 192.

Moreover, wound healing can be further accelerated by genetically modified stem cell exosomes 49, 193-195. Conversely, not all stem cell exosomes promote wound healing. BMMSC-exo, while promoting cell proliferation, does not affect angiogenesis and wound healing 57. However, BMMSC-exo pretreated with deferoxamine activates the PI3K/AKT signaling pathway through the miR-126-mediated down-regulation of PTEN, thereby promoting angiogenesis and wound healing in diabetic rats 58. These studies provide a new idea for cell-free therapy to treat diabetic chronic wounds.

#### Eczema

Eczema is a skin inflammatory reaction caused by various internal and external factors accompanied by severe itching. There is currently no cure for eczema; treatment and life management can relieve itching and prevent a recurrence. Disturbances in immune regulation affect wound healing, possibly causing hypertrophic scarring or inhibiting tissue regeneration 196, 197. Treatment of eczema combined with wounds is a tedious process. Fortunately, exosomes have both the functions of regulating immunity and promoting wound healing. Studies based on an eczema wound model showed that hucMSC-exos accelerated wound healing in mice by inhibiting inflammatory cell infiltration and promoting angiogenesis 198, demonstrating the pleiotropic advantage of stem cell exosomes in the treatment of complex wounds.

#### Other chronic wounds

In addition to these complex wounds, many other chronic wounds are poorly studied, such as venous leg ulcers and pressure ulcers. Persistent inflammation is an important feature of chronic wounds. It is manifested by increased enzymatic activity, dysregulated cytokines, suppressed angiogenesis, reduced wound re-epithelialization, and an imbalance in the proportion of immune cells 199. Stem cell exosomes have excellent effects in restoring cell function and promoting tissue regeneration. It is likely to be a potential drug for addressing these chronic wounds.

### The development direction of engineered exosomes

#### Engineered exosomes

After all, the exosome is a carrier, and the internal substrates play their biological role. To improve the therapeutic effect of exosomes, we can change the substrates and their levels. The most common approach is to increase substrate concentration by genetically engineering overexpression, such as knock-in/knock-out DNA fragments and transfer plasmids. For example, synovial mesenchymal stem cells (SMSCs) can only promote fibroblast proliferation but not angiogenesis. It's known that miR-126 promotes angiogenesis by enhancing the expression of fibroblast and vascular endothelial growth factors. Thus, by upregulating miR-126-3p levels through gene overexpression technology, SMSCs can simultaneously facilitate fibroblast proliferation and angiogenesis, resulting in accelerated diabetic wound healing 28. Besides miRNA, increasing the content of growth factors is also an effective way.

Another approach is to pretreat exosome-producing cells to alter exosome properties. Several studies have shown that the pretreatment of MSCs with drugs, cytokines, and physical factors can enhance the effect of MSCs and their exosomes in promoting tissue regeneration 71, 200-207. The pretreatment method is technically more straightforward, safer, and more universal than genetically engineered exosomes. Therefore, engineered exosomes are likely to become a hot spot in the treatment of wounds in the future.

#### Drug-loaded materials

After the wound is healed, a closed scab will be formed, making it difficult for drugs to penetrate. As a drug carrier, exosomes usually exist for a short time. Making exosomes play a long-term and stable effect can solve the problem of sustained drug release. Fortunately, advances in materials science have provided solutions to these difficulties. For example, chitosan is widely used for drug delivery and accelerating wound closure, but its ability to promote the recovery of sub-epidermal tissue is not good enough 208. When making a chitosan hydrogel wound dressing containing exosomes, the dressing contained the dual properties of both chitosan and exosomes. Both the speed and quality of wound healing have been improved 28, 209. Moreover, more ingredients can be added to the composite to enhance the therapeutic effect further.

#### Composite materials

**Figure 5:**
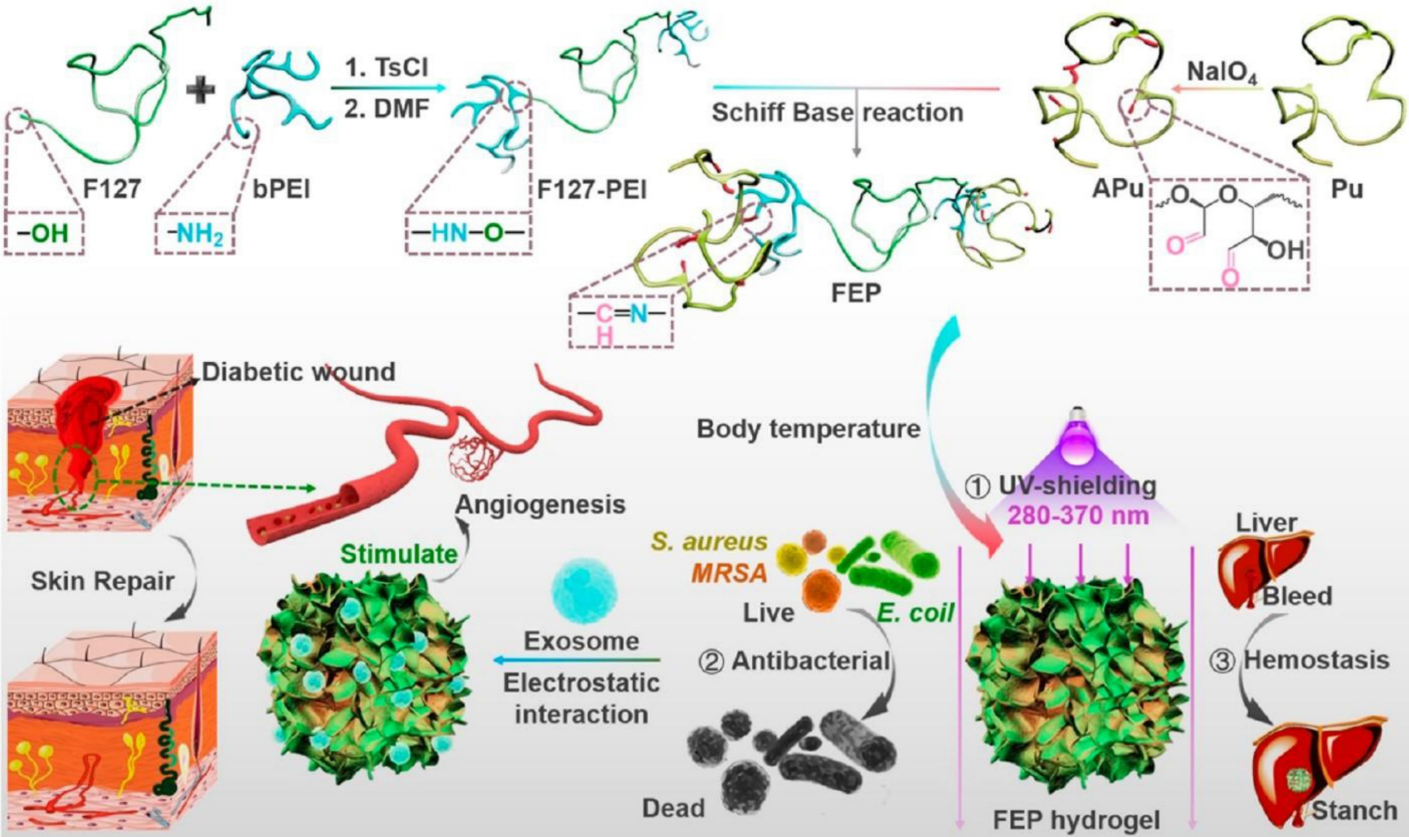
**Schematic diagram of an exosome-combined multifunctional dressing for diabetic wound repair.** This study synthesized a composite material containing F127-PEI, APu, Multifunctional FEP Scaffold Dressing, and exosomes. The dressing has multifunctional properties such as efficient antibacterial/resistant bacteria, rapid hemostasis, self-healing, tissue adhesion, and good UV shielding properties. This provides a typical research idea for the application of engineered exosomes in complex wound repair. This picture is reprinted with permission from 211. Copyright 2022 American Chemical Society.

The drug-loaded material is suitable for ordinary wounds, and the effect on complex wounds needs further improvement. For example, in diabetic ulcers, it is difficult for ordinary drug-loaded materials to promote their healing. The exosome-containing composite materials for such wounds are the most numerous. Shiekn et al. have developed a material for promoting the healing of diabetic ulcers 210. The material contains various components and functions, including a gel matrix that promotes cell migration, an elastomeric antioxidant polyurethane (PUAO) that attenuates oxidative stress, a calcium peroxide-PUAO cryogels that continuously releases oxygen, and ADCS-exos that promote tissue angiogenesis and collagen expression. In addition to these ingredients, antibacterial agents and growth factors can be added to increase efficacy further (160) (figure 5). There are many similar studies and materials on why diabetic ulcers are difficult to heal. Mixing a variety of substances allows multiple effects, and finally, the purpose of the diabetic wound is achieved.

#### Targeting therapy

Exosomes are mainly used as a dressing for wound treatment. However, for complex wounds such as burns, complications can also be alleviated by intravenous injection of exosomes. How to improve its targeted therapy ability is a way to improve efficacy and reduce the dosage. The current methods to improve the targeting of exosomes include combining with materials and using exosomes derived from special cells 212-214. For example, using macrophage-derived exosomes as carriers to deliver drugs to lesion areas 215. It mainly exploits the properties of injured tissue to release chemokines and cytokines to recruit macrophages for aggregation.

In addition, exosomes can be used to deliver genetic, western, and traditional Chinese medicines, reducing the number of drugs and side effects 216-218. However, certain tissues, such as the blood-brain barrier, prevent drugs from penetrating, reducing drug efficacy. Using the homing features of macrophages to combine the modification of homing peptides can increase the exosome homing and penetration ability 122, 219.

#### Responsive biomaterials

Irregular wounds reduce the fit of the dressing and thus reduce the drug's effectiveness. One study suggested that thermosensitive materials, such as Pluronic F-127 hydrogel in liquid form at low temperature and semi-solid gel at high temperature, could improve dressing fit 220. The therapeutic capacity was effectively enhanced by mixing Pluronic F-127 hydrogel with exosomes. Compared with the exosome or hydrogel treatment, the mixed dressing significantly improved the healing speed of diabetic ulcers. In addition, pH, light, electricity, ultrasound, and magnetic field-responsive biomaterials have all been developed, providing directions for future responsive composite development 221.

### The potential application of exosomes in wounds

**Figure 6:**
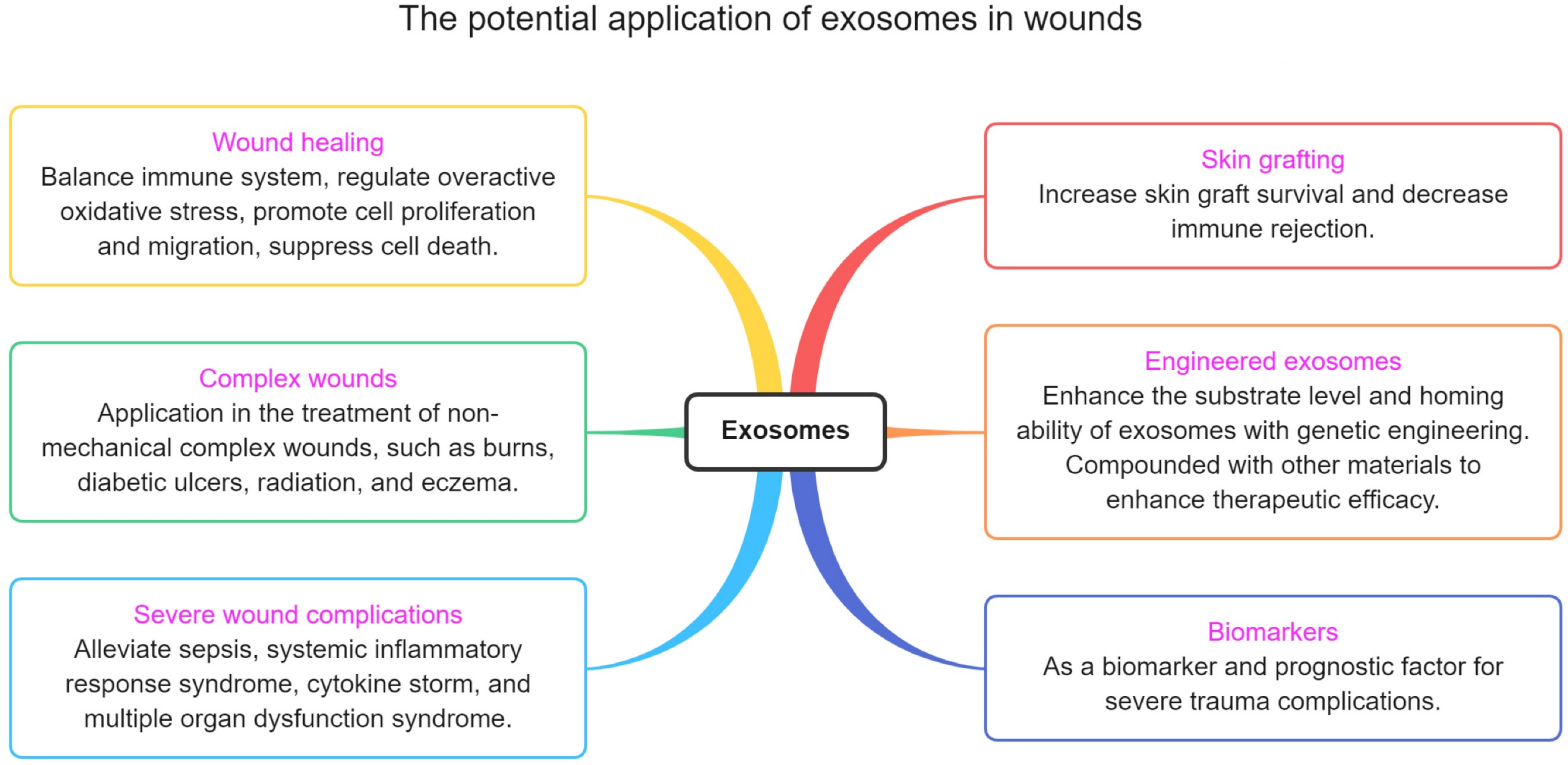
The potential application of exosomes in wounds.

Exosomes not only can be used to promote wound repair, but there are also many trauma-related points that have not been taken seriously (figure 6). This section describes these potential applications.

#### Skin graft

Skin grafting is a common technique for wound treatment. Although technologies such as micro-skins and artificial skins have been developed to reduce the risk of insufficient donor skin in patients, poor graft survival and immune rejection remain critical issues 222. Recent studies have confirmed that stem cells and their exosomes can effectively increase the survival rate of transplanted organs and decrease the foreign body response 13, 223-225. However, these studies are more focused on the role of exosomes in promoting tissue regeneration, and there are still few studies on immune responses. Modified exosomes could boost skin transplant survival by lowering immunological rejection. However, only a few studies provided evidence of that 226, 227. Undoubtedly, reducing immune rejection by injecting or modifying exosomes may become a key to the future treatment of patients who require skin grafts.

#### Complications

Clinically, the treatment of small-area trauma is relatively easy. The difficulty lies in treating severe trauma and its complications. Because of the lack of a skin barrier system, severe trauma patients often risk sepsis/systemic inflammatory response syndrome (SIRS)/cytokine storm due to infection 228, 229. If the infection worsens, patients will likely die from multiple organ dysfunction syndromes 230. In the course of treatment, antibacterial and dialysis are the keys to preventing the onset of SIRS. Thanks to the development of these two technologies, the incidence of SIRS in patients with severe trauma and burns has been greatly reduced. Despite this, recent studies have shown that patients are still more likely to develop internal organ failure after discharge than the general population 231, which may be related to persistent inflammation 232, 233. Therefore, it is necessary to find new drugs to balance immune regulation, both in terms of short-term and long-term treatment.

The role of stem cells and their exosomes in regulating the systemic immune system has been widely recognized and is an extremely promising drug 234. Unfortunately, exosomes and engineered exosomes have been poorly studied in trauma complications. We draw on other studies to discuss the potential value of exosomes in trauma complications. For example, MSC-exos can modulate the lung immune microenvironment by inhibiting the expression of pro-inflammatory factors, promoting the expression of anti-inflammatory factors, and balancing the differentiation of T cells, thereby reducing SIRS and lung injury caused by infection 235-237. Acute kidney injury (AKI) is also a common complication of sepsis. Tubular epithelial cell-derived exosomes (exosomal miRNA-19b-3p) reduce LPS-induced AKI by suppressing inflammatory factors expression and macrophage M1 subtype activation 238. Furthermore, due to the COVID-19 epidemic, stem cells and their exosomes' role in treating SIRS has gained widespread attention 239. Numerous studies have confirmed the role of exosomes in diagnosing and treating SIRS. It can be seen that the mechanism of exosomes in the treatment of SIRS-induced multiple organ failure involves anti-inflammatory, antioxidant, and regulation of key gene expression to inhibit cell damage and promote tissue regeneration.

#### Biomarkers

Severe trauma has the potential to cause multiple complications or exacerbate pre-existing conditions. Exosomes are extremely promising targets for liquid biopsy diagnostics 240. For example, in LPS-induced liver injury, LPS elevates the expression of exosomal miR-103-3p by activating macrophages, thereby leading to liver fibrosis by activating KLF4 241. Similarly, abnormal levels of exosomal miRNAs in patients with sepsis are associated with liver injury, circulatory injury, and central nervous system injury 234. It indicates that healthy exosomes have therapeutic effects, while patient-derived exosomes can be used to diagnose sepsis complications. Thus, in some cases, exosomes can be used as biomarkers for diagnosing trauma complications. At present, there are few studies in this area, and it is also a potential research direction.

### Clinical studies of exosome therapy for wounds, related diseases, and applications

All of the above presentations illustrate exosomes' diagnostic and therapeutic potential in wounds, complex wounds, and associated complications. Exosomes demonstrate good potential for clinical application, having similar efficacy to that of derived cells providing an alternative means of cell-free therapy. It has to be said that stem cell clinical therapies are only taking shape, with more products in the pipeline but few being registered by the government for filing. And exosomes, as a new product, are still in their early stages. There are many animal studies and few clinical studies. There are not many references in wound therapy.

There are fewer clinical studies on wound treatment. For example, Kwon et al. demonstrated that ADSC-exos significantly improved the results of CO_2_ laser treatment of acne scars, including reduced scarring, post-treatment pain, dryness, edema, and erythema 242. Exosomes have been studied not infrequently in treating visceral injuries but remain limited. Perhaps due to ethical and safety considerations, more exosome-related clinical studies have focused on biomarkers and drug carriers. This suggests that there are still many research fields that deserve to be explored.

In contrast, exosomes are more frequently used as biomarkers in clinical studies. Wang et al. found that miRNAs of exosomes in the peripheral blood circulation of kidney transplant recipients with delayed graft function were altered, with hsa-miR-33a-5p_R-1, hsa-miR-98-5p, and hsa-miR-151a-5p being significantly upregulated 243. Sun et al. found that urinary exosomal CD63 levels were significantly elevated in diabetic nephropathy patients with early kidney injury and then significantly declined after treatment with alpha-lipoic acid, suggesting not only that CD63 in urinary exosomes could be a biomarker of efficacy but also a potentially important therapeutic target 244.

Another exciting study demonstrates the feasibility of exosomes as delivery vehicles. Baier et al. found that exosomes in milk prevented the degradation of substrate miRNAs, thus delivering them into the blood of the drinker and thus exerting physiological effects on the body 245. Two other studies found that nebulised ADSC-exos and hucMSC-exos could treat COVID-19-induced pneumonia 246, 247. These studies provide new references for the role of exosomes and the mode of delivery.

### Potential adverse effects and challenges of exosomes in clinical application

Stem cell exosomes are considered a safe therapy, but this concern has not been substantiated in preclinical and clinical trials. Some studies still have found adverse shreds of evidence and raised concerns. For example, the safety of the donor source of the exosome, the population for which the recipient is suitable, the immunogenicity of the allogeneic cells, the quality control of manufacture, and the cost of production. This section will discuss these issues and suggest possible solutions.

#### Tumorigenic risk

Although exosomes, as a non-cellular substance, do not directly transform into tumors, this does not mean that exosomes do not risk promoting tumor formation. For example, multiple myeloma (MM)-BMMSC exosomes promote MM tumor cell growth, whereas normal BMMSC exosomes inhibit MM tumor cell growth *in vivo* and *in vitro* 248. MM-BMMSC exosomes are also found to accelerate tumor cell dissemination and metastasis. Gastric cancer-based studies have shown that stem cell exosomes have the potential to accelerate tumor growth and angiogenesis 249.

Conversely, another study has yielded the exact opposite results 250. All of these studies show that the source and target of exosomes determine their safety in clinical use. It should be ensured that exosomes' source and application subjects are not cancer patients. At the same time, testing standards/biomarkers for the safety assessment of tumorigenesis should be developed.

#### Immunogenicity

Immunogenicity is an unavoidable topic in biotherapy. Many allogeneic transplant recipients have had to use immunosuppressants for long periods to reduce immune activity, which has affected the body's normal immune system while ensuring the survival of the graft. As stem cells have become better understood, it has been found that MSCs derived from umbilical cords generally have low immunogenicity, which is why umbilical cord-derived MSCs are currently the most recorded product for clinical research. Theoretically, exosomes have low immunogenicity, primarily when derived from hucMSC, which have already low immunogenicity. Therefore, when designing drugs, researchers preferentially select cells with low immunogenicity as the source of exosomes. Fortunately, at present, we have not heard that there is a severe problem of immune rejection in the study of exosomes.

However, low immunogenicity does not mean that it is not present. For example, membrane proteins from cells in the exosome membrane, conformation, glycosylation and polymorphism of proteins in the substrate, impurities and stabilisers in production, and route and frequency of administration. It requires producers to establish complete production and quality control standards and analyze immunogenicity under government authorities' requirements.

#### Production standards and quality control

The biological functions of exosomes are achieved by transporting the substrates they contain, such as nucleic acids, proteins, and lipids. If the contained substance deviates from the normal level, it may cause unpredictable effects. For example, the plasma of pulmonary embolism patients contains up-regulated microRNA-28-3p 251, which was also detected in serum exosomes 252. If exosomes contain up-regulated microRNA-28-3p, it will aggravate the apoptosis of pulmonary endothelial cells 253. In addition, exosomes may cause untargeted cell growth. For example, inoculation of BMMSCs into infarcted myocardium induces cardiac sympathetic nerve sprouting 254. BMMSCs exosomes may also have similar adverse effects. These studies suggest that stem cell exosomes require extensive preclinical evaluation before clinical application.

In addition, cell senescence is inevitable in culture. Generally speaking, stem cells will decline in physiology and function after 3-5 generations of culture, and the substrate of exosomes will also change. To avoid these impacts, manufacturers need to establish production standards and quality control systems, which not only avoid excessive variables during production but also ensure the stability of the final products.

#### Cost

Side effects are an inevitable topic for all medical technologies, but the cost is the real key to their popularity. The issue of cost control is currently present with exosomes.

The first issue is the source of the cells. The efficacy of normal cells constantly decays in culture, and the substrate and effectiveness of the secreted exosomes change accordingly. The efficacy of normal cells constantly decays in culture, and the substrate and efficacy of the secreted exosomes vary accordingly. For this reason, new sources of cells, such as umbilical cords, have to be found repeatedly. This process is labour-intensive, time-consuming, and costly regarding raw materials.

The second issue is cell culture and exosome isolation. Currently, the cost of cell culture is relatively stable, and the main problem is that the yield of exosomes is too low. Repeated cell cultures are too costly if sufficient quantities of exosomes are to be obtained for therapeutic use.

A third issue is a preservation. Although exosomes prevent substrate degradation, they are still inherently unstable and can be stored for a minimal time after secretion. To ensure efficacy, freshly harvested exosomes are often used in studies. This limitation also requires that the place of production is not too far away from the site of use to avoid any loss of efficacy during transportation.

#### Solutions

Bioengineering techniques can solve all the mentioned issues. Bioengineering can be used not only to modify cells and exosomes but is also an excellent approach to maintaining stable production systems. For example, genetic modification techniques are used to generate regular immortalised cells (cell factories), which avoids the difficulties of cell source, cell stability, and immunogenicity, and also allows for higher yields and lower costs. In addition, three-dimensional cell culture systems are gradually maturing, facilitating the consistent and stable manufacture of exosomes and can further reduce costs. On the other hand, enterprises need to develop proper production standards and quality control systems, and regulators need to develop relevant laws and regulations.

### Prospectives of exosome-based wound healing

In the medical discipline of traumatology, poor healing of cutaneous wounds is a prevalent issue. The utilization of standard therapeutic modalities, such as chemical molecule therapeutics and conventional dressings, has not been able to produce adequate results due to the complex pathophysiological processes of wound healing. In recent years, conclusive data has demonstrated that exosomes offer significant therapeutic potential for wound healing and regeneration. The overall aim of exosome utilization is to obtain clinical therapeutic benefits; however, there is still a long way to go. Firstly, the inadequate generation of exosomes is the most significant issue. Conventional methods for extracting and purifying exosomes are highly complicated. Some newly discovered approaches, such as EXODUS 255, can provide rapid, high-purity, and high-yield extraction of exosomes from biofluids. Similarly, more efficient procedures must be developed for exosomes' high-purity and high-yield extraction. Secondly, there are no standard criteria for vesicle size, purity, contamination levels, or the detection of particular biomarkers. Exosomes have not been subjected to a proteome study, nor have strategies been established to differentiate exosomes depending on the cell source. Last but not least, safety must be the foundation of all clinical applications. However, research evaluating the safety of exosome-based therapeutics is still in its infancy, and numerous questions remain unanswered. Exosomes require research to establish the appropriate dosage range for safe clinical use.

MSC-exos have demonstrated favorable effects on cutaneous wound healing in animal studies and pre-clinical trials; however, evidence from clinical research on exosomes and cutaneous wound healing is currently insufficient. Excitingly, several meta-analyses suggest that MSC-exos are a prospective and promising treatment for numerous acute and chronic disorders, including cutaneous wounds in pre-clinical investigations 256-258, highlighting the anti-inflammatory and anti-trauma properties of MSC-exos. This increases the likelihood of a successful clinical translation of MSC-exos for cutaneous wound healing. The fact that acne scars treated with human ADSC-exos and fractional CO2 laser showed superior improvement than the control-treated group in a randomized double-blind controlled clinical study by Kwon et al. provided further evidence that ADSC-exos had synergistic therapeutic effects on clinical therapies for atrophic acne scars 242. As a result, the potential for MSC-exos to be successfully translated into clinical practice is promising.

Improving the therapeutic effectiveness of MSC-exosomes is a goal to pursue once they have been implemented in clinical settings. Using exosomes in conjunction with biomaterials is one strategy for achieving a synergistic effect. Exosomes play a crucial role in nearly every wound healing and repair step. For instance, they control inflammatory responses, encourage cell migration and proliferation, increase angiogenesis, and control ECM remodelling. A recent study by Wang and colleagues described using methylcellulose-chitosan hydrogels loaded with exosomes to treat severe wounds under diabetic conditions 259. The hydrogels created an environment conducive to cell growth and ECM remodelling by acting as three-dimensional porous scaffolds. Specifically, based on the hydrogels, exosomes might be released sustainably over a lengthy period of time and exert long-lasting therapeutic effects for improved efficacy. Exosomes can be used in a wider variety of contexts because of the versatility afforded by biomaterial transformation. For example, when applied to diabetic wound models, MSC-exosome in combination with hydrogel and adhesive UV shielding exosome-releasing dressing produced more significant therapeutic benefits on wound healing and skin reconstruction 211, 220. Despite this, the therapeutic implementation of hydrogel-based delivery methods *in vivo* still confronts several obstacles. For instance, leftover crosslinkers or hydrogel components might have side effects that need further study. It is possible for pH- or temperature-sensitive hydrogels to undergo uncontrollable cross-linking, which can block the needle during injection. To prevent early gel formation, it is vital to adjust the conditions under which the gel develops. Moreover, the *in vitro* established release patterns of hydrogel-based delivery methods may not be appropriate for *in vivo* applications. It is still necessary to investigate the impact of the *in vivo* microenvironment on delivery efficiency.

Exosome characteristics, such as their contents or the activities of their surface molecules, can also be bioengineered. The targeted therapeutic molecules (such as miRNAs or therapeutics) can be loaded into exosomes in order to provide exogenous effectiveness 194. Additionally, the surface of exosomes can be changed with functional molecules, such as aptamers, to facilitate the transfer of altered exosomes to target areas when supplied systemically or locally, hence enhancing therapeutic efficacy. Combining the aforementioned systems will improve exosomes' therapeutic effectiveness in cutaneous wound healing and regeneration.

## Conclusions

In conclusion, exosomes have shown a pivotal role in promoting wound healing and skin regeneration by inducing the release of anti-inflammatory, antioxidant, anti-apoptotic, and pro-angiogenic mediators. Meanwhile, exosomes are promising to replace stem cells as a new generation of biological drugs. Moreover, exosome therapy has emerged as a pioneering cell-free treatment option, avoiding the issues of the immediate utility of MSCs. However, the scale of production and cost, preservation, and delivery strategies limit their clinical application. Further, there is an urgent need to define robust, reliable efficacy tests to determine the efficacy of exosome-based therapies.

## Figures and Tables

**Table 1:** Exosome isolation methods.

Isolation technique	Principle	Processing time	Advantages	Purity	Recovery	Reference
Differential Centrifugation	Sedimentation rate	3-6 h	• Low cost • Simple operation • Suitable for large-volume	Low	5-20%	126, 127
Density gradient ultracentrifugation	Density, size and shape	20-24 h	• Simple operation	High	10-40%	126
Precipitation methods	Sedimentation rate	2-18 h	• High throughput • Simple operation	Low	5-30%	128
Ultrafiltration	Size	1-3 h	• Simple operation • Portability • Capable of operating with low amount of sample • Low equipment cost	Moderate	~30%	127, 129
Size-exclusion chromatography	Size	1-2 h	• Intact structure of isolated exosomes • High selectivity	Moderate	40-80%	129, 130
Immunoaffinity	Surface marker expression	18-20 h	• High purity and selectivity • Specific exosomes isolation	Very high	>90%	131
Microfluidics-based techniques	Surface marker expression, size, density, etc.	1-3 h	• Providing higher resolution and sensitivity	High	40-90%	132
